# Validation of the Parkinson Fatigue Scale in Chinese Parkinson's disease patients

**DOI:** 10.1002/brb3.712

**Published:** 2017-05-02

**Authors:** Rao Fu, Shi‐Shuang Cui, Juan‐Juan Du, Pei Huang, Ya‐Chao He, Chao Gao, Xiao‐Guang Luo, Sheng‐Di Chen

**Affiliations:** ^1^ Department of Neurology & Institute of NeurologyRuijin Hospital Affiliated to Shanghai Jiao Tong University School of MedicineShanghaiChina; ^2^ Department of NeurologyFirst Affiliated Hospital of China Medical UniversityShenyangChina

**Keywords:** fatigue, Parkinson's disease, questionnaire, reliability, validity

## Abstract

**Objective:**

Fatigue is a common nonmotor symptom in Parkinson's disease (PD); however, the Parkinson's disease fatigue scale (PFS), which is designed to measure fatigue in PD, has not been validated in China. The aim of this study was to determine the validity and reliability of the Chinese version of the PFS in PD patients.

**Methods:**

A total of 115 PD patients were evaluated at baseline and after 7 days. Assessments included the PFS, the Fatigue Severity Scale (FSS), and scales assessing motor, cognition, depression, and anxiety. Acceptability was assessed in terms of the rate of missing data and floor and ceiling effects. Cronbach's alpha was calculated to determine internal consistency. Test–retest reliability was assessed using the intraclass correlation coefficient (ICC). Spearman's rank correlation coefficients were used to calculate convergent and divergent validity between PFS scores and scales assessing clinical characteristics.

**Results:**

No data were missing for the PFS. Compared with the original scoring method, the binary scoring method had relatively large floor effects (5.21% vs. 17.39%) and ceiling effects (0.90% vs. 4.31%). The internal consistency and test–retest reliability of the PFS were satisfactory (original scoring method: Cronbach's alpha = 0.97, ICC = 0.94; binary scoring method: Cronbach's alpha = 0.94, ICC = 0.94). The PFS score exhibited strong convergent validity with FSS score (correlation coefficient = 0.87). PFS score was weakly to moderately correlated with disease duration and with measures of disease stage, motor function, depression, and anxiety (range of correlation coefficients: 0.25–0.48). There was no significant correlation between PFS score and either onset age or MoCA score (range of correlation coefficients: −0.05 to 0.12).

**Conclusion:**

The Chinese version of the PFS is a valid measure for assessing fatigue in PD.

## 1 Introduction

Parkinson's disease (PD), the second most common neurodegenerative disease, is clinically characterized by motor symptoms, such as tremor, rigidity, and akinesia (Kalia & Lang, 2015). Traditionally, most research has focused on the motor symptoms of PD. In the past decade, however, attention has shifted to the many nonmotor symptoms (NMS) in PD, including fatigue (Kluger et al., 2016). Currently, a lack of consensus exists regarding a precise definition of PD fatigue. PD patients describe their fatigue as a “feeling of abnormal and overwhelming tiredness and lack of energy that is distinct both qualitatively and quantitatively from normal tiredness” (Brown, Dittner, Findley, & Wessely, 2005). According to a recent study, fatigue is one of the most bothersome symptoms associated with PD (Uebelacker, Epstein‐Lubow, Lewis, Broughton, & Friedman, 2014). In addition, several studies have shown that fatigue exists in over half of PD patients in China (Fu, Luo, Ren, He, & Lv, 2016; Zuo et al., 2016). Importantly, fatigue is a leading cause of disability in PD patients and dramatically compromises their daily living activities and quality of life (Alves, Wentzel‐Larsen, & Larsen, 2004; Stocchi et al., 2014).

Despite the enormous impact and relatively large prevalence of fatigue in PD, little progress has been made in understanding its etiology or pathophysiology or to develop effective clinical treatment methods (Elbers, Berendse, & Kwakkel, 2016; Friedman, Abrantes, & Sweet, 2011; Friedman et al., 2007). One major barrier could be the lack of an appropriate instrument to measure fatigue in PD patients. In the absence of a gold standard to assess fatigue, the most prevalent method of assessing fatigue is through self‐report rating instruments. In 2005, Brown and associates developed a brief and easy to complete scale, called the Parkinson's disease Fatigue Scale (PFS), specifically for use in PD patients and confirmed its validity and reliability (Brown et al., 2005). In addition, the PFS is available in several languages (Grace, Mendelsohn, & Friedman, 2007; Hagell, Rosblom, & Palhagen, 2012; Kummer, Scalzo, Cardoso, & Teixeira, 2011). However, the psychometric properties of the PFS have not been evaluated in Chinese PD patients. The aim of this study was to determine the validity and reliability of the PFS in Chinese PD patients.

## 2 Methods

### 2.1 Design

This was a cross‐sectional, one‐point‐in‐time evaluation with retest study.

### 2.2 Patients

Consecutive patients were recruited from the Movement Disorders Clinic at the Department of Neurology at Ruijin Hospital, which is affiliated with Shanghai Jiao Tong University School of Medicine (Shanghai, China), during the period from December 2015 to June 2016. All patients fulfilled the Movement Disorder Society (MDS) clinical diagnostic criteria for PD (Postuma et al., 2015). Patients were excluded if they were unable to complete the questionnaires due to poor comprehension or were unable to cooperate when undergoing a complete neurological examination. A total of 115 patients were included in the study to assess the validity of the PFS. All patients enrolled in the study gave written informed consent, and the study was approved by the Ethics Committee of Ruijin Hospital, Shanghai Jiao Tong University School of Medicine.

### 2.3 Procedure

Demographic data were collected for all enrolled PD patients. The total amount of dopaminergic medication was expressed as the levodopa equivalent daily dosage (LEDD), which was determined using previously reported methods (Tomlinson et al., 2010). Patients were assessed at baseline in the “ON” state via a comprehensive evaluation (including motor symptoms and NMS) and after 7 days (time range, 5–9 days). The 7‐day assessment, which consisted of only the PFS, was conducted by telephone. During the 7‐day follow‐up, all the enrolled patients received medication.

### 2.4 Assessments

#### 2.4.1 Fatigue

To assess the validity and reliability of the PFS, we obtained a linguistically validated version of the PFS in simplified Chinese from Dr. Richard G. Brown. The PFS was adapted to simplified Chinese using the translation/retranslation method. Briefly, the forward translation of PFS into Mandarin was performed by two consultants with excellent knowledge of Chinese, and an initial PFS version was developed by consensus. Then, a backward translation of the consensus version into English was performed by two other independent consultants, and the back‐translated version was modified to eliminate discrepancies between the original and the back‐translated version. Finally, a preliminary test was conducted in five PD patients to evaluate the appropriateness and the comprehensibility of PFS in simplified Chinese. The PFS is a 16‐item self‐reported scale that is specifically designed to assess the physical aspects of fatigue in PD patients (Brown et al., 2005). Two scoring methods exist for the PFS. In the original method, the item response options range from 1 (*strongly disagree*) to 5 (*strongly agree*). The total PFS‐16 score ranges from 1 to 5 and is obtained by dividing the sum of all item scores by 16. In the binary scoring method, the item responses are dichotomized into 1 and 0 (*agree* and *strongly agree* are scored as 1, all other responses are scored as 0). The method yields a total score between 0 and 16 (16 = more fatigue) (Brown et al., 2005).

The Fatigue Severity Scale (FSS) (Krupp, LaRocca, Muir‐Nash, & Steinberg, 1989) is a nine‐item scale that covers a time frame of the past 2 weeks. Patients are asked to rate how each item describes their fatigue level from 1 (*strongly disagree*) to 7 (*strongly agree*). The total FSS score represents the mean score of each of the nine items, with scores ranging from 1 to 7, and a higher score indicates a higher level of fatigue. The cultural adaptation and validation of the FSS has been conducted in Chinese patients with major depressive disorder and in nondepressive people (Wang, Liu, Chiu, & Tsai, 2016). It is the only scale to receive a “recommended” rating from the MDS Task Force for both screening and severity in PD patients (Friedman et al., 2010).

#### 2.4.2 Other measures

Motor symptoms were evaluated using the Movement Disorder Society‐sponsored revision of the Unified Parkinson's disease Rating Scale (MDS‐UPDRS) (Goetz et al., 2007). Modified Hoehn and Yahr (H–Y) staging was used to stage PD patients (Hoehn & Yahr, 1967). NMS were evaluated using the following scales: the Montreal Cognitive Assessment (MoCA) Beijing version for cognitive function (the test form and instructions are available at the official MoCA website http://www.mocatest.org/), the 17‐item Hamilton Depression Scale (HAMD) for depression (Hamilton, 1960; Zheng et al., 1988), and the 14‐item Hamilton Anxiety Scale (HAMA) for anxiety (Hamilton, 1959). For some of the rating scales (H–Y stage, MDS‐UPDRS, HAMA, and HAMD), a higher score indicated higher severity of the construct being measured, whereas for the remainder of the scales, a higher score indicated the opposite.

### 2.5 Data analysis

The patients’ demographic and clinical data are presented as descriptive statistics. The measurement data are reported as the means ± standard deviations (*SD*). In addition to the use of descriptive statistics to define the sample, the clinimetric attributes were assessed as follows:

1. Acceptability: Acceptability was assessed using not only the rate of missing data but also floor (the proportion of patients with the minimum possible score) and ceiling (the proportion of patients with the maximum possible score) effects. Missing data rates <5% were considered acceptable (Smith et al., 2005). The floor/ceiling effects were required to be <15% (Ambrosio et al., 2016). The mean and median difference was considered acceptable at less than 10% of the maximum observed value. The limits for skewness were −1 and +1 (Hays, Anderson, & Revicki, 1993).
2. Reliability: The reliability of the PFS was evaluated using the internal consistency reliability and test–retest reliability. For internal consistency, Cronbach's alpha (0.80 or higher was considered acceptable) and the corrected item‐total correlation (an item‐total correlation ≥0.40 was considered acceptable) were computed. To assess the test–retest reliability, the PFS was repeated after 7 days (time range, 5–9 days), and an intraclass coefficient (ICC) between the baseline and the 7‐day assessment was calculated for each item and the total score (an ICC of 0.70 or higher was considered acceptable) (Ware & Gandek, 1998).
3. Validity: To examine convergent validity, Spearman's rank correlations were used to evaluate the correlations between the PFS total score and the FSS score. Divergent validity was evaluated using Spearman's rank correlations between PFS total score, anxiety (HAMA), depression (HAMD), and cognition (MoCA). In addition, associations between total PFS score and the following constructs were determined: H–Y staging, MDS‐UPDRS scores overall and for each subscale, demographic information (age, onset age, education). Significant correlation coefficients that were >0.5 were interpreted as strong, coefficients of 0.3–0.5 were interpreted as moderate, and coefficients less than 0.3 were interpreted as weak (Terwee et al., 2007).

All analyses were conducted using SPSS software (version 20.0), and the level of significance in all analyses was *p *<* *.05.

## 3 Results

### 3.1 Sample characteristics

The demographic and clinical profiles of the patients are presented in Table 1. The study included 65 men and 50 women, and the overall mean age of the participants was 62.83 ± 9.55 years, with a mean disease duration of 5.34 ± 4.47 years. Most of the patients were in the early H–Y stage. The mean MDS‐UPDRS total score was 49.44 ± 28.74, which indicated mild to moderate impairment.

**Table 1:** Demographic and clinical characteristics of the sample

Variable	Mean (*SD*)
Age (years)	62.83 (9.55)
Onset age (years)	57.48 (10.16)
Gender (male/female)	65/50
Education level, cases/total (%)	
Primary school and below	20/115 (17.39)
Middle and high school	56/115 (48.69)
Bachelor's degree and above	39/115 (33.91)
Disease duration (years)	5.34 (4.47)
H–Y stage	1.770 (0.68)
Stage 1	34/115 (29.57%)
Stage 1.5	21/115 (18.26%)
Stage 2	36/115 (31.30%)
Stage 2.5	16/115 (13.91%)
Stage 3	7/115 (6.08%)
Stage 4	0/115 (0%)
Stage 5	1/115 (0.87%)
LEDD (mg/day)	343.42 (319.42)
MDS‐UPDRS I	9.68 (5.85)
MDS‐UPDRS II	11.27 (7.06)
MDS‐UPDRS III	26.95 (18.33)
MDS‐UPDRS IV	1.54 (3.17)
MDS‐UPDRS total	49.44 (28.74)
MoCA	21.76 (4.84)
HAMD	5.98 (4.54)
HAMA	7.56 (6.06)
PFS	2.81 (1.06)
FSS	3.53 (1.88)

H–Y stage, modified Hoehn and Yahr staging; LEDD, levodopa equivalent daily dosage; MDS‐UPDRS, Movement Disorder Society‐sponsored revision of the Unified Parkinson's Disease Rating Scale; MoCA, Montreal Cognitive Assessment; HAMD, 17‐item Hamilton Depression Scale; HAMA, 14‐item Hamilton Anxiety Scale; PFS, Parkinson Fatigue Scale; FSS, Fatigue Severity Scale.

Data are expressed as numbers, with percentages in parentheses or as mean ± *SD*.

### 3.2 Acceptability

No data were missing for the PFS. For the original scoring method, the floor effect was 5.21%, and the ceiling effect was nearly negligible (0.90%), both the floor effect and the ceiling effect were below the standard limits. Compared to the original scoring method, a relatively large floor effect (17.39%) and ceiling (4.31%) effect were observed when the binary method was used. The difference between the mean and median PFS scores (both with the original scoring method and the binary scoring method) was less than 10% of the maximum observed value, and the skewness was also acceptable.

### 3.3 Reliability

For the original scoring method, the Cronbach's alpha for the PFS total score was 0.97 (Table 2), and for the binary method, the Cronbach's alpha was 0.94 (Table 2). The test–retest reliability (ICC) for the total score was 0.94 and therefore was sufficiently high (Table 2). The ICC values for items ranged from 0.74 to 0.85, which also indicated high test–retest reliability (Table 2).

**Table 2:** Descriptive and reliability statistics for scores of the Chinese version of the PFS

PFS items	Original scoring method (1–5)				Binary scoring method (0 or 1)			
	Mean (*SD*)	Corrected item‐total correlation	Alpha if item deleted	ICC	Mean (*SD*)	Corrected item‐total correlation	Alpha if item deleted	ICC
1. I have to rest during the day	3.13 (1.20)	0.62	0.97	0.74	0.55 (0.50)	0.57	0.94	0.72
2. My life is restricted by fatigue	2.97 (1.26)	0.87	0.96	0.81	0.47 (0.50)	0.82	0.93	0.79
3. I get tired more quickly than other people I know	3.51 (1.36)	0.75	0.96	0.80	0.64 (0.48)	0.73	0.93	0.75
4. Fatigue is one of my three worst symptoms	2.37 (1.27)	0.71	0.96	0.81	0.24 (0.43)	0.55	0.94	0.71
5. I feel completely exhausted	2.48(1.28)	0.78	0.96	0.81	0.27 (0.45)	0.67	0.93	0.69
6. Fatigue makes me reluctant to socialize	2.40 (1.24)	0.68	0.96	0.84	0.24 (0.43)	0.59	0.94	0.71
7. Because of fatigue it takes me longer to get things done	3.20 (1.41)	0.82	0.96	0.83	0.57 (0.50)	0.75	0.93	0.77
8. I have a feeling of “heaviness”	3.16 (1.31)	0.73	0.96	0.80	0.58 (0.50)	0.58	0.94	0.73
9. If I wasn't so tired I could do more things	3.14 (1.32)	0.81	0.96	0.83	0.54 (0.50)	0.69	0.93	0.76
10. Everything I do is an effort	2.64 (1.30)	0.81	0.96	0.85	0.32 (0.47)	0.64	0.93	0.74
11. I lack energy for much of the time	2.80 (1.31)	0.84	0.96	0.85	0.43 (0.50)	0.72	0.93	0.72
12. I feel totally drained	2.61 (1.28)	0.83	0.96	0.80	0.32 (0.47)	0.71	0.93	0.66
13. Fatigue makes it difficult for me to cope with everyday activities	2.50 (1.31)	0.80	0.96	0.80	0.31 (0.47)	0.65	0.93	0.63
14. I feel tired even when I haven't done anything	2.65 (1.34)	0.83	0.96	0.82	0.36 (0.48)	0.68	0.93	0.71
15. Because of fatigue I do less in my day than I would like	2.96 (1.38)	0.85	0.96	0.76	0.50 (0.50)	0.80	0.93	0.69
16. I get so tired I want to lie down wherever I am	2.41 (1.27)	0.74	0.96	0.80	0.26 (0.44)	0.63	0.94	0.67
PFS average/total score	2.81 (1.06)	Cronbach's alpha = 0.97		0.94	6.59 (5.47)	Cronbach's alpha = 0.94		0.94

*SD*, standard deviation; ICC, intraclass correlation coefficient; PFS, Parkinson Fatigue Scale.

Data are expressed as means ± *SD*.

### 3.4 Validity

The correlations of the average PFS score to the other variables in the present study are shown in Table 3. A significant correlation was found between the PFS score and the FSS score (*r* = .87, *p *<* *.05), which demonstrates good convergent validity. The PFS average score increased as the H–Y stage increased (*r *= .24, *p *=* *.01).

**Table 3:** Correlations between PFS score and other clinical characteristics

	Spearman's rank correlation	*p* value
Age	0.23	.01^a^
Education (years)	0.04	.70
Onset age	0.12	.21
Disease duration (years)	0.24	.01^a^
LEDD (mg/day)	0.25	.01^a^
H–Y stage	0.24	.01^a^
MDS‐UPDRS I	0.48	<.05^a^
MDS‐UPDRS II	0.37	<.05^a^
MDS‐UPDRS III	0.20	.02^a^
MDS‐UPDRS IV	0.29	<.05^a^
MDS‐UPDRS total	0.35	<.05^a^
MoCA	−0.05	.53
HAMD	0.42	<.05^a^
HAMA	0.39	<.05^a^
FSS	0.87	<.05^a^

H–Y stage, modified Hoehn and Yahr staging; LEDD, levodopa equivalent daily dosage; MDS‐UPDRS, Movement Disorder Society‐sponsored revision of the Unified Parkinson's Disease Rating Scale; MoCA, Montreal Cognitive Assessment; HAMD, 17‐item Hamilton Depression Scale; HAMA, 14‐item Hamilton Anxiety Scale; PFS, Parkinson Fatigue Scale; FSS, Fatigue Severity Scale.

a Significant difference.

The correlations between the PFS score and the psychiatric measures were moderate (HAMD: *r *= .42, *p *<* *.05; HAMA: *r *= .39, *p *<* *.05). PFS score was weakly to moderately correlated with disease duration, disease severity (MDS‐UPDRS scores overall and for each subscale), and the LEDD. PFS score was not significantly correlated with either onset age or MoCA score (Table 3).

## 4 Discussion

This is the first study to investigate the psychometric properties of the Chinese version of the PFS. Our observations suggest that this version exhibits good reliability and validity.

Regarding the reliability, as shown in Table 2, the internal consistency of the Chinese version of the PFS was satisfactory (Cronbach's alpha = 0.94–0.97) for both scoring methods (original scoring method and binary scoring method). The 16 items composing the PFS were significantly correlated with the total scores (*r *= .57–.87), which indicated a single coherent construct. Test–retest reliability is a meaningful assessment to evaluate the stability of a scale. Therefore, we calculated the ICC between the baseline and the 7‐day assessment for the individual items and the total PFS score. In contrast to the findings of Brown et al. (2005) that the ICC value of the overall PFS score obtained using both the original score method and the binary method was 0.82–0.83, our study indicated a more robust ICC value of 0.94. This discrepancy may be due to the different test–retest intervals in the two studies. In Brown et al.'s (2005) study, patients completed the retest after an approximately 2‐week period, which may account for some of the differences in the results obtained in the two studies. However, both our results and the results from the Brown's study suggest that the overall PFS score exhibits reasonable reliability.

Regarding convergent validity, the correlation between the PFS score and FSS score was analyzed. The FSS is a widely used measure of fatigue that fulfills the criteria of a “recommended” fatigue scale for PD (both for screening and severity). We found a strong correlation between the scores of the PFS and FSS, which indicated good convergent validity of the scale. Previous studies conducted on PD patients have also reported similar findings (Brown et al., 2005; Grace et al., 2007; Hagell et al., 2012; Kummer et al., 2011). Moreover, the PFS score increased as the H–Y stage increased, which indicated a satisfactory discriminative validity. The correlations between disease duration, stage, or motor symptoms and fatigue are still controversial (Fabbrini et al., 2013). In the present study, we found a significant correlation between fatigue and the above‐mentioned clinical characteristics (including disease duration and motor symptoms), which indicated that dysfunction of dopamine may be involved in the pathogenesis of fatigue in PD patients. Further studies are needed to clarify the details of these correlations. The close relationship between anxiety, depression, and fatigue is widely recognized (Fu et al., 2016; Solla et al., 2014; Stocchi et al., 2014) and is consistent with our findings.

Consistent with previous results (Friedman et al., 2011; Hagell et al., 2012; Kummer et al., 2011), the quality of the data obtained in the present study was satisfactory, and we had no missing data. Our study found floor/ceiling effects in both scoring methods, specifically that the floor effect was relatively large for the binary scoring method (17.39%, which was beyond the acceptable level for the floor effect). Consistent with our results, Hagell et al. (2012) found that for the binary scoring method, scaling assumption tests were not particularly convincing, with relatively large floor effects observed. However, Brown et al. (2005) reported no floor/ceiling effects for either scoring methods. One possible explanation for this discrepancy is the different composition of the samples in the different studies. Importantly, a comparison of our patients with the patients examined by Brown et al. (2005) revealed that our patients were younger (62.8 years vs. 70.4 years) and had a shorter disease duration (5.8 years vs. 7.9 years); these factors may have affected the performance of the PFS. A second explanation for the discrepancy between the studies could be due to the binary scoring method itself, which could lead to a loss of information and precision (Hobart & Cano, 2009). It is therefore worth noting that the application of the binary scoring method should be used with caution in clinical practice, despite its ease of use.

This study has several limitations. One aspect that was not evaluated here was content validity. Additionally, the results were from a single center, and most of the PD patients resided in the eastern region of China, which is not representative of PD patients throughout China. Therefore, future studies should validate the PFS using a large population of PD patients in China recruited from across multiple centers. Third, no age‐ and sex‐matched controls were included in the study design; therefore, we were unable to calculate the sensitivity and specificity or establish a cut‐off score for the PFS, which should be established in future studies. Nevertheless, in light of these limitations, we propose that the Chinese version of the PFS is a valid and reliable instrument to assess fatigue in PD patients. The PFS can be used in clinical trials to better understand fatigue among PD patients in China.

## Conflicts of Interest

None declared.
